# Precise size-matching between guest polyoxometalates and host metal-organic frameworks enables enhanced photocatalytic water oxidation

**DOI:** 10.1038/s42004-025-01838-y

**Published:** 2025-12-10

**Authors:** Waqas Ali Shah, Xusheng Dai, Xiaowei Zhai, Yuanyuan Zhao, Yalei Zhang, Shujun Li

**Affiliations:** School of Chemistry and Chemical Engineering, Henan Key Laboratory of Boron Chemistry and Advanced Materials, Key Laboratory of Green Chemical Media and Reactions, Ministry of Education, Collaborative Innovation Centre of Henan Province for Green Manufacturing of Fine Chemicals, Henan Normal University, Xinxiang, China

**Keywords:** Metal-organic frameworks, Porous materials, Photocatalysis, Hydrogen energy

## Abstract

The development of robust and efficient heterogeneous photocatalysts for water oxidation is a significant challenge in solar fuel production. Polyoxometalate-metal-organic framework composites (POM@MOFs) represent a promising platform, yet achieving optimal electronic interaction remains a key goal. We demonstrate that electronic communication between polyoxometalates (POMs) and metal-organic frameworks (MOFs) enhances charge transfer kinetics while suppressing electron-hole recombination, with maximum efficiency achieved through precise size-matching between MOF cavities and encapsulated POMs. This principle is illustrated by Co4@UiO-67 (C1), where encapsulation of Na₁₀[(PW₉O₃₄)₂Co₄(H₂O)₂] in UiO-67’s cavities creates a leaching-proof composite. This confinement prevents aggregation, yielding excellent photocatalytic water oxidation performance (283 TONs)—surpassing prior Co4@MOF systems. Mechanistic insights from photoluminescence reveal efficient charge separation, while post-catalytic analysis confirms structural integrity and reusability. Overall, this work introduces a new paradigm in the design of POM@MOF composites, positioning C1 as a robust, recyclable, and highly active photocatalyst for heterogeneous water oxidation.

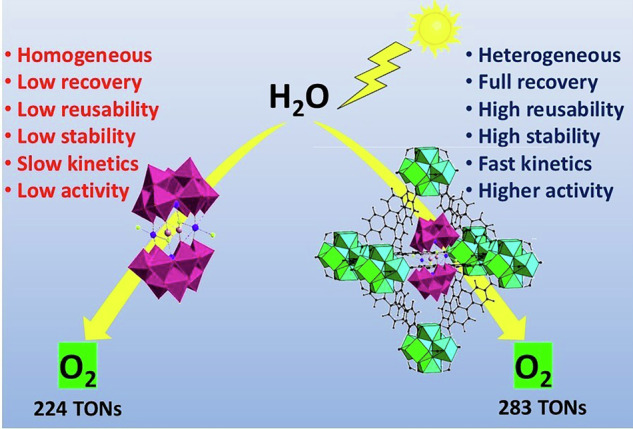

## Abstract (web summary)

Efficient charge transfer and reduced electron-hole recombination are critical challenges in photocatalytic systems. Here, the authors demonstrate that precise size-matching between guest polyoxometalates and host metal-organic frameworks, exemplified using Co4@UiO-67, enhances photocatalytic water oxidation owing to accelerated electron transfer and suppressed charge recombination.

## Introduction

Polyoxometalates (POMs) are molecular metal-oxo clusters composed of corner- and edge-sharing metal–oxygen polyhedra, often considered homogeneous analogs of bulk metal oxides^1^. Functioning as electron reservoirs, they exhibit exceptional redox properties, allowing reversible oxidation–reduction processes without structural degradation, and have been widely employed as catalysts across various fields^2,3^. However, their homogeneous nature presents several limitations, including poor recoverability and recyclability, low structural and chemical stability, limited surface area, and a tendency to agglomerate^4^. Metal-organic frameworks (MOFs), on the other hand, are highly porous, crystalline polymeric materials built from metal ions or clusters bridged by organic linkers^5,6^. The integration of POMs into the porous cavities of MOFs not only immobilizes these otherwise soluble catalysts but also effectively addresses all the aforementioned limitations of POMs. This encapsulation introduces synergistic effects, resulting in multifunctional materials with enhanced or even emergent properties^7,8^.

Polyoxometalate embedded metal-organic frameworks (PMOFs)-originally employed to heterogenize POMs, have since evolved into advanced platforms that combine the redox versatility and acidity of POMs with the high surface area, tunable pore size, and structural diversity of MOFs^9,10^. The synergy arises from non-covalent electrostatic interactions—either unidirectional or bidirectional electron transfer—between the two components, supported by optimal size-confinement and orbital overlap. This interaction often results in significant improvements in catalytic activity, chemical and thermal stability, pH tolerance, and light absorption behavior (e.g., bathochromic and hyperchromic shifts)^7,11,12^. Despite the lack of covalent bonding, both components retain their identities, yet their properties are amplified. In some cases, the composite even exhibits a catalytic behavior not present in either component alone^13^. Such POM–MOF composites have demonstrated broad utility in supercapacitors, organic transformations, heavy metal adsorption, dye degradation, sensors, batteries, antimicrobial activity, and electrocatalysis, including HER and OER^10^.

Among these, Na₁₀[(PW₉O₃₄)₂Co₄(H₂O)₂] (Co4)—a noble-metal- and carbon-free cobalt-based Well-Dawson POM—is a particularly promising water oxidation catalyst due to its tetranuclear Co core sandwiched between two PW₉ units. However, its practical application is limited by low surface area, poor stability, and difficulty in recovery, all arising from its homogeneous nature. Previous attempts to heterogenize Co4 in MOFs have yielded mixed results. For instance, Co₄@MIL-101(Cr) demonstrated improved activity but suffered from significant leaching due to the MOF’s window sizes exceeding the ~18 Å diameter of Co4^14^. In the case of Co₄@MOF-545, the porphyrin ligands acted as built-in photosensitizers, eliminating the need for an external PS; however, severe leaching remained a major issue, undermining the purpose of heterogenization through encapsulation^15^. Conversely, Co₄@MIL-100(Fe) effectively prevented leaching and showed a modest improvement in activity, though still relatively limited^16^. These examples highlight an apparent trade-off between stability and performance, suggesting that the catalytic activity of Co4 can be tuned by altering the MOF support.

Effective design of PMOF assemblies requires precise size-matching between the POM and the MOF host—not only in cavity dimensions but also in aperture (window) size. A MOF with cavity windows smaller than the POM in all directions prevents leaching, while a cavity size that closely matches the POM enhances electronic communication through improved contact area and electron transfer kinetics^11^. Oversized cavities weaken confinement and hinder charge transfer, whereas oversized windows lead to POM leaching. On the other hand, undersized cavities may obstruct substrate diffusion and suppress catalytic activity. Thus, optimal performance arises from a delicate balance: tight confinement without blocking accessibility, enabling stable, leaching-resistant assemblies with enhanced catalytic function^17^.

To resolve these issues, we encapsulated Co4 into UiO-67, a Zr(IV)-based MOF featuring octahedral (21 Å) and tetrahedral (11.5 Å) pores, accessible through ~8 Å windows—smaller than the size of Co4^18^. This tight confinement not only prevents leaching but also improves electron transfer kinetics via increased contact area, while the inherent chemical and thermal robustness of UiO-67 further supports catalyst integrity^19^. Additionally, the supercages of UiO-67 offer sufficient space for effective encapsulation, and the narrow windows suppress POM aggregation while allowing substrate diffusion^20^. Thus, UiO-67 plays a dual role: ensuring permanent immobilization of Co4 and facilitating improved photocatalytic performance. Its structure supports spatial isolation of POMs, preventing aggregation, while promoting fast and directional charge transfer. Electrons generated during water oxidation are transferred efficiently to both the sacrificial electron acceptor (SEA) and the MOF itself, enhancing catalytic turnover^11,21,22^.

The resulting composite, Co4@UiO-67 (C1), was fully characterized and exhibited permanent POM encapsulation with zero leaching. This encapsulation strategy not only ensures leaching-free behavior but also addresses the long-standing challenge of catalyst degradation observed in other POM@MOF systems, such as Co4@MIL-101(Cr) and MOF-545, where leaching significantly reduces catalytic performance over time. C1 demonstrated enhanced thermal and chemical stability and superior light-harvesting properties. What sets this work apart is the size-matched encapsulation of Co4 within UiO-67 (C1), which optimizes electron transfer kinetics, reduces electron-hole recombination, and significantly enhances catalytic performance over a broader pH range compared to previous Co4-based composites. This precise design not only improves charge separation and electron mobility but also increases activity, as shown by time-resolved and steady-state photoluminescence studies. This work presents a leaching-resistant, recyclable noble-metal-free photocatalyst platform, advancing sustainable energy conversion by enhancing the efficiency, stability, and reusability of water oxidation catalysts. C1 maintained its catalytic activity and structural integrity across multiple recycling cycles, demonstrating its robustness for heterogeneous water oxidation.

## Results and discussion

### Characterization

Composite C1, consisting of Co4 encapsulated in UiO-67, was synthesized using the “bottle-around-ship” (BAS) method with varying POM loadings in UiO-67, ranging from 0.15 to 0.23 POMs per MOF. Among these, the composite with 0.21 POMs per MOF was selected for further characterization and experimentation, as it exhibited the highest catalytic activity Table S1. This observation aligns with well-established reports showing that increasing the POM content in MOFs enhances catalytic activity up to a certain threshold, beyond which additional POM loading leads to a decrease in performance^23–27^.

The successful permanent encapsulation of Co4 in UiO-67 is attributed to the framework’s smaller window size compared to the dimensions of the POM^28^. Previous studies have demonstrated that POMs preferentially occupy the larger octahedral cages (~21 Å) in UiO-67, and the framework’s flexibility allows it to effectively adjust its pore structure to accommodate such bulky guests^17,29^.

**Fig. 1:**
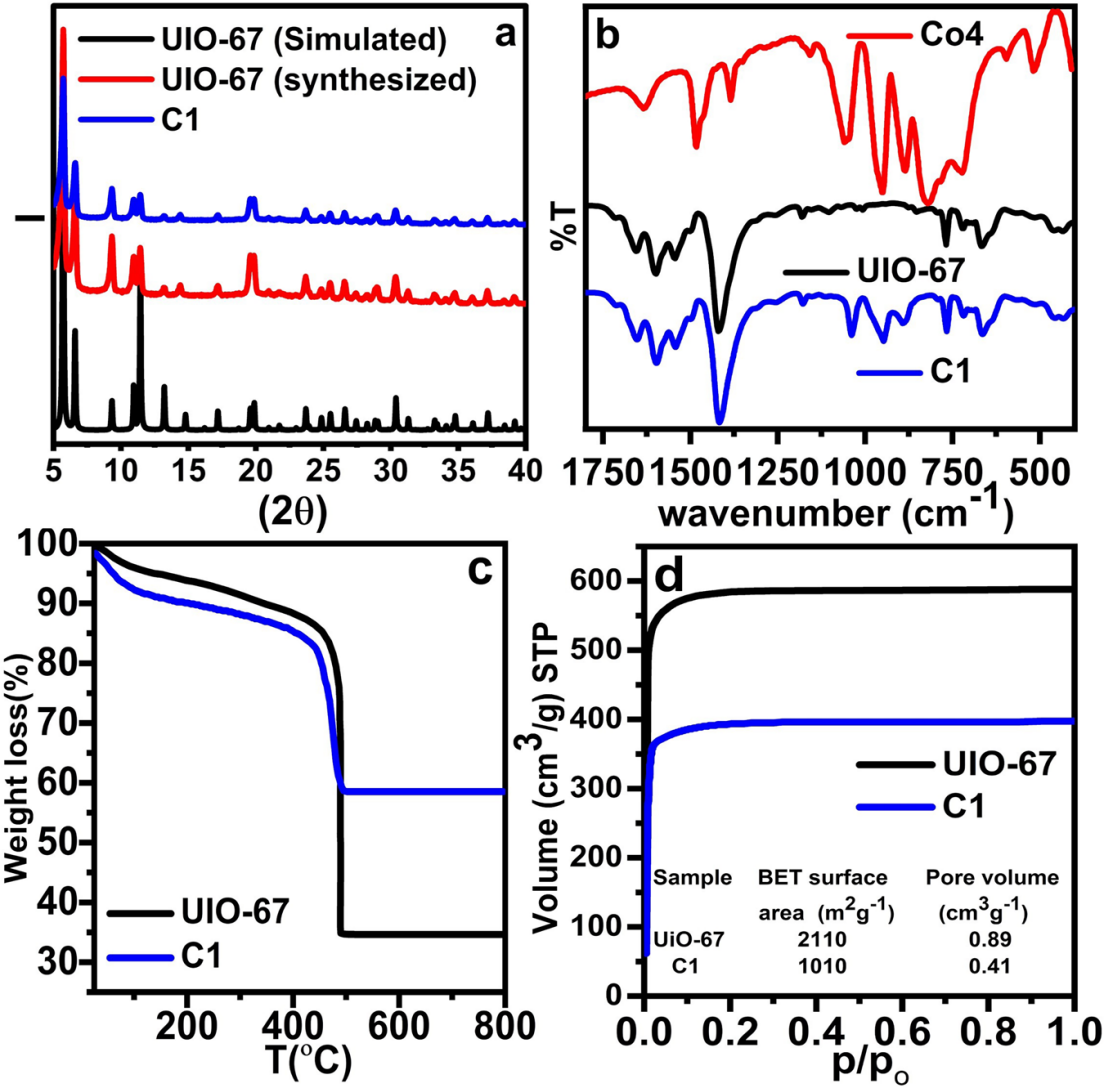
Various characterizations of Co4, UiO-67, and C1. **a** PXRD of UiO-67 and C1. **b** FTIR of Co4, UiO-67 and C1. **c** TGA results of UiO-67 and C1. **d** N_2_ Adsorption isotherms of UiO-67 and C1.

Figure 1a shows the PXRD pattern of composite C1 alongside the simulated pattern of UiO-67^19^. The close match between the experimental and simulated patterns confirms that the crystalline structure of UiO-67 remains intact following POM encapsulation. Additionally, the absence of diffraction peaks corresponding to Co4 indicates that the POM is not present on the external surface but rather encapsulated within the MOF cavities. Since POMs do not form crystalline phases inside confined MOF cavities, they typically exhibit no PXRD peaks^30,31^. This necessitates the use of complementary techniques such as FTIR and ICP to confirm encapsulation.

The FTIR spectra of UiO-67, Co4, and composite C1 (Fig. 1b) further verify the successful encapsulation. Characteristic peaks of Co₄ are clearly observed in the composite, with only minor shifts toward higher wavenumbers, which can be attributed to electrostatic interactions between POMs and the MOF framework^14^. This also indicates that the MOF structure remains largely unchanged during the encapsulation process^19,32^.

Elemental analysis results for UiO-67, Co4, and C1 are presented in Table S2. The close agreement between experimental and theoretical values confirms compositional reliability. The absence of Na⁺ in the composite suggests that the POMs reside within the MOF as either protonated or anionic species. The number of acidic protons was quantified by acid–base titration^33^. Thermogravimetric analysis (TGA) of UiO-67 and composite C1 (Fig. 1c) reveals distinct differences. UiO-67 exhibits two major weight loss steps at 120 °C (removal of adsorbed water) and at 480 °C (decomposition of organic ligands), consistent with literature reports. In contrast, composite C1 shows a significantly reduced total weight loss (41.5%) compared to pristine UiO-67 (65.2%), corroborating the presence of non-volatile POMs within the MOF. The higher final decomposition temperature in C1 also suggests enhanced thermal stability due to encapsulated POMs^17,34^. Compositional formulas of UiO-67, Co4, and C1 were deduced using data from proton titration, ICP, and TGA, and are detailed in Table S3^35^.

Nitrogen adsorption isotherms (Fig. 1d, Table S4) were used to determine the surface area via the Brunauer–Emmett–Teller (BET) method. The BET surface area of UiO-67 decreased from 2110 to 1010 m^2^ g^−1^, and the pore volume reduced from 0.89 to 0.41 cm^3^ g^−1^ upon the formation of composite C1. Additionally, the pore size distribution (PSD) profiles of UiO-67 and C1 (Fig. S1) further confirm the effective encapsulation and occupation of the pores after Co4 encapsulation^17,36^.

The optical absorption behavior of the materials was investigated using UV–vis diffuse reflectance spectroscopy (DRS) (Fig. S2). UiO-67 shows characteristic bands at 379, 436, 516, 550, 596, and 654 nm, while Co4 exhibits peaks at 375 and 561 nm^34^. The spectrum of C1 features absorptions corresponding to both components, with noticeable red shifts—indicative of strong electronic interactions. A prominent hyperchromic shift observed in C1 further suggests enhanced light harvesting due to the synergistic interaction between POM and MOF components^16^.

**Fig. 2:**
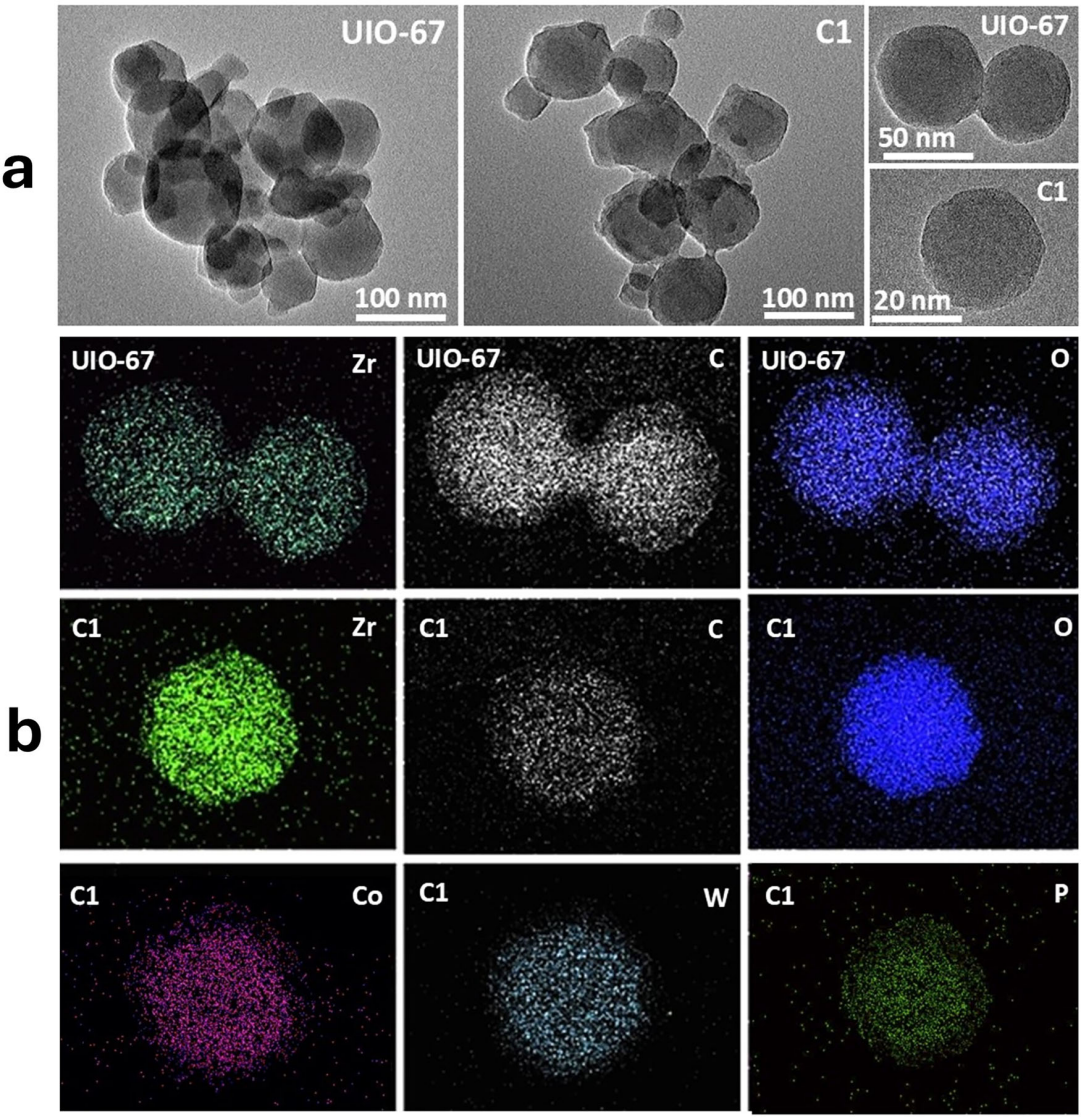
TEM and elemental mapping. **a** TEM images of UiO-67 and composite C1 at various resolutions, confirming the integrity of the structure after encapsulation. **b** EDS elemental mapping of UiO-67 and C1 showing the homogeneous distribution of various elements.

Transmission electron microscopy (TEM) and elemental mapping (Fig. 2) reveal that UiO-67 crystallites retain their uniform square-like morphology with slightly rounded corners after encapsulation. The structural uniformity in composite C1 confirms that the integrity of the MOF framework is preserved^37^. Furthermore, the presence of only one type of particle morphology indicates effective encapsulation, rather than surface adsorption or physical mixing. Elemental mapping via EDX shows a homogeneous distribution of elements from both the POM and MOF, supporting deep encapsulation.

**Fig. 3:**
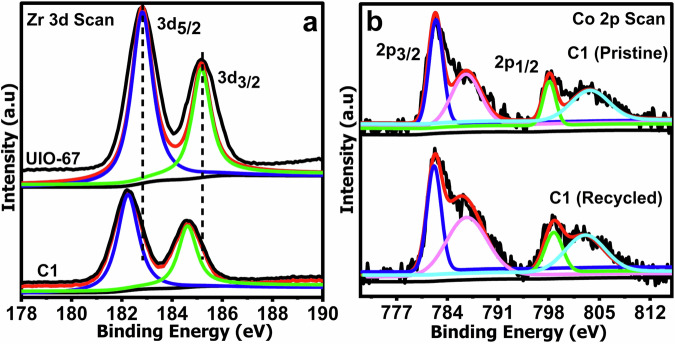
XPS of Co4, UiO-67 and C1. **a** Zr in UiO-67 and C1, **b** Co in Co4 and C1.

X-ray photoelectron spectroscopy (XPS) analyses of UiO-67, Co4, and C1 (Figs. 3 and S3) further confirm the intact oxidation states of all elements before and after encapsulation. The Zr 3d peaks in UiO-67 appear at 182.7 and 185.1 eV (Fig. 3a), and show a slight shift to lower binding energies in C1—suggesting electron density transfer from the POM to the MOF^38^. All POM elements were only detected after Ar sputtering, implying that POMs are embedded within MOF cavities rather than surface-bound^39^. The Co 2p spectrum of C1 (Fig. 3b) features main peaks at 780.5 and 796.5 eV with satellites at 787 and 802.9 eV, appearing at slightly higher binding energies compared to pure Co4, indicating a net electron donation from Co4 to the UiO-67 framework. Similar shifts were observed in the W 4f spectra (Fig. S3a), further supporting the charge redistribution hypothesis^40^. The P 2p peak appeared at 134 eV (Fig. S3b), consistent with previous reports^41^. Decreased signal intensities of certain elements in the composite also indicate lower elemental percentages due to dilution upon encapsulation.

### Photocatalytic activities

The photocatalytic activities of Co4 and its composite C1 were evaluated through a series of experiments under visible light irradiation across a pH range of 8–7. Fresh catalyst samples were used in each experiment, and reactions were carried out under optimized conditions.

**Fig. 4:**
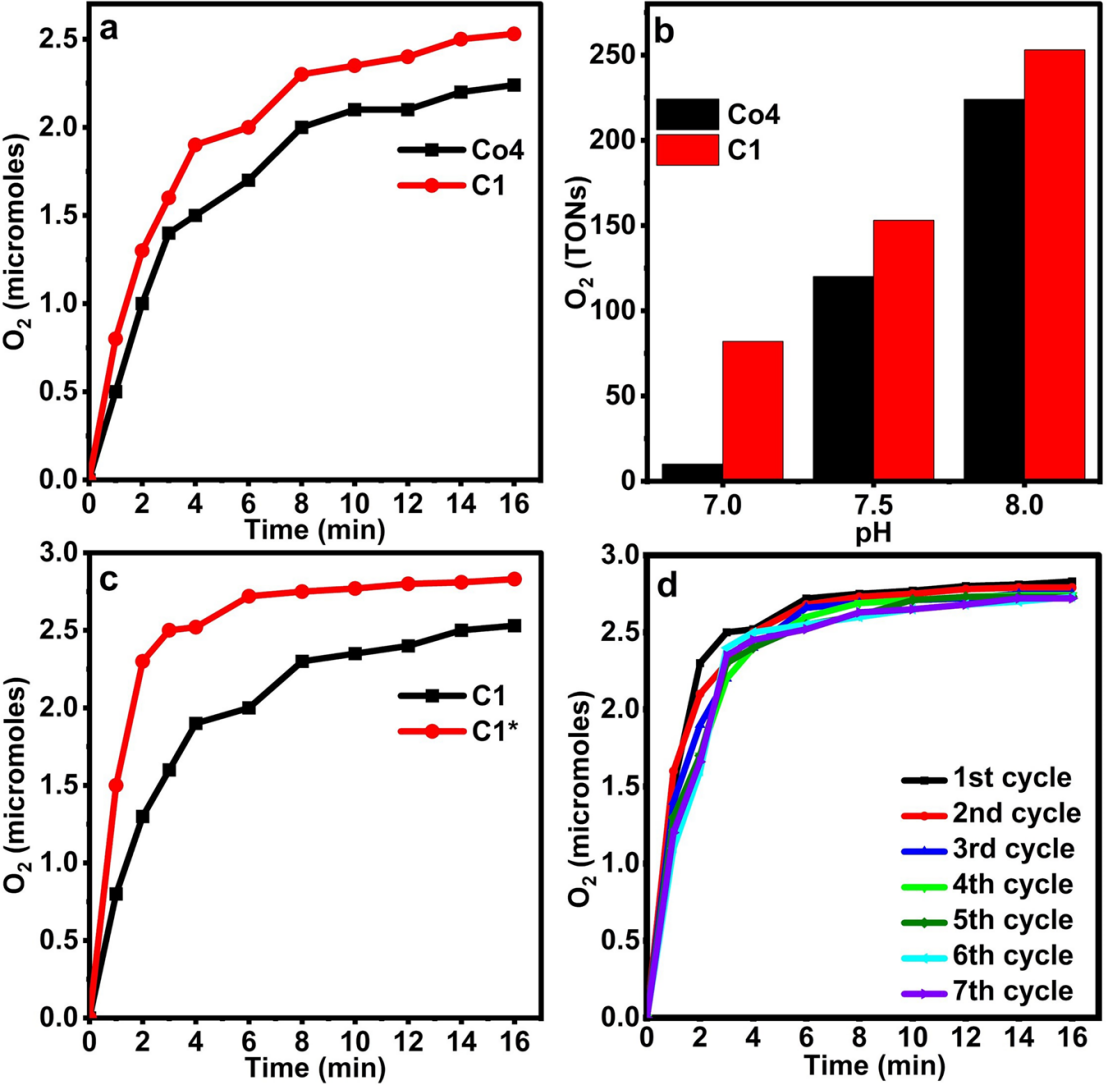
Comparison of the OER activity of homogeneous and encapsulated Co4. **a** OER activity of Co4 and C1 at pH 8 and **b** Effect of pH on OER activity of Co4 and C1 under identical conditions*^a^. **c** Comparison of OER activity of C1 under identical conditions to Co4*^a^ and under newly optimized conditions*^b^ with new optimized conditions*^b^ and **d** Recycling experiments under new optimized conditions*^b^. *^a^5 µM catalyst’s concentration, with 5 mM SEA, and 1 mM PS in 30 mM borate buffer at various pH. *^b^6.5 µM catalyst’s concentration, with 7 mM SEA, and 1.2 mM PS in 30 mM borate buffer at various pH.

Figure 4a, b present the OER performance of Co4 and C1 at varying pH levels (8–7.5 in borate buffer, and pH 7 in deionized water), using Na₂S₂O₈ as the sacrificial electron acceptor (SEA) and [Ru(bpy)₃]Cl₂·6H₂O as the photosensitizer (PS). At pH 8, Co4 generated 2.24 μmol of O₂ and a TON of 224. When the pH was decreased to 7.5, these values dropped to 1.2 μmol and TONs of 120. Under neutral conditions (pH 7), OER activity was completely suppressed. These findings are consistent with previously reported results^42^.

In contrast, encapsulating Co4 within UiO-67 led to a significant enhancement in photocatalytic performance. Under identical conditions, C1 produced 2.53 μmol of O_2_ and a TON of 253 at pH 8—outperforming comparable systems such as Co₄@MIL-101(Cr), MIL-100(Fe), and MOF-545. This enhancement aligns with established literature noting improved POM activity upon immobilization within MOFs^15^. Even at pH 7.5, C1 retained notable activity (1.53 μmol), and unlike free Co4, C1 remained catalytically active at neutral pH, generating 0.82 μmol of O_2_. This indicates that encapsulation not only increases activity but also broadens the operational pH range. Notably, these experiments were conducted under conditions previously optimized for Co4^42^; further optimization for C1 is necessary, as POMs exhibit different behavior when confined within MOF frameworks.

The influence of catalyst, SEA, and PS concentrations on OER activity was systematically studied to identify optimal conditions for C1. As shown in Fig. S4, increasing the C1 concentration from 2 μM to 6.5 μM led to a rise in O_2_ evolution from 0.7 to 2.83 μmol. Beyond this point, further increases in catalyst concentration had negligible effects. Figure S5 illustrates the impact of SEA concentration. Increasing SEA from 2 to 7 mM resulted in a rise in OER activity from 2.41 to 2.77 μmol, beyond which further increases showed no significant improvement. This plateau suggests saturation behavior. Similarly, increasing the PS concentration from 0.5 to 1.2 mM boosted OER from 2 to 2.83 μmol, after which the reaction rate plateaued (Fig. S6). This tunability in PS concentration offers a distinct advantage over systems with built-in photosensitizers. Figure 4c compares the OER performance of C1 under previously reported (Co4-optimized) and newly optimized conditions, clearly demonstrating the benefits of tailored optimization.

The long-term catalytic performance of C1 was also evaluated (Fig. S7). O_2_ evolution increased steadily, reaching a plateau after 1 h, and remained stable for an additional 2.5 h. A subsequent decline in activity was observed, which was restored by replenishing the SEA (adding 7 mM Na₂S₂O₈), confirming that activity loss was due to SEA depletion rather than catalyst deactivation. Such behavior, including extended stability of heterogenized Co₄ over a period of one month, is consistent with previous studies^15,43^.

C1’s reusability was validated through recycling experiments. As shown in Fig. 4d, C1 retained its OER performance over seven consecutive cycles, highlighting its excellent stability. This sustained activity is attributed to the permanent, leaching-resistant encapsulation of Co4 within UiO-67, contrasting with other MOFs where catalytic performance declines due to leaching in subsequent cycles^15^. This remarkable consistency is also observed in several other POM@UiO-67 systems, which maintain their catalytic activity over multiple cycles^20,44,45^.

### Mechanistic studies

To elucidate the improved electron transfer dynamics in C1 compared to Co4, we conducted a systematic study using luminescence quenching and time-resolved photoluminescence spectroscopy to measure excited-state lifetimes.

### Photoluminescence (PL) quenching

**Fig. 5:**
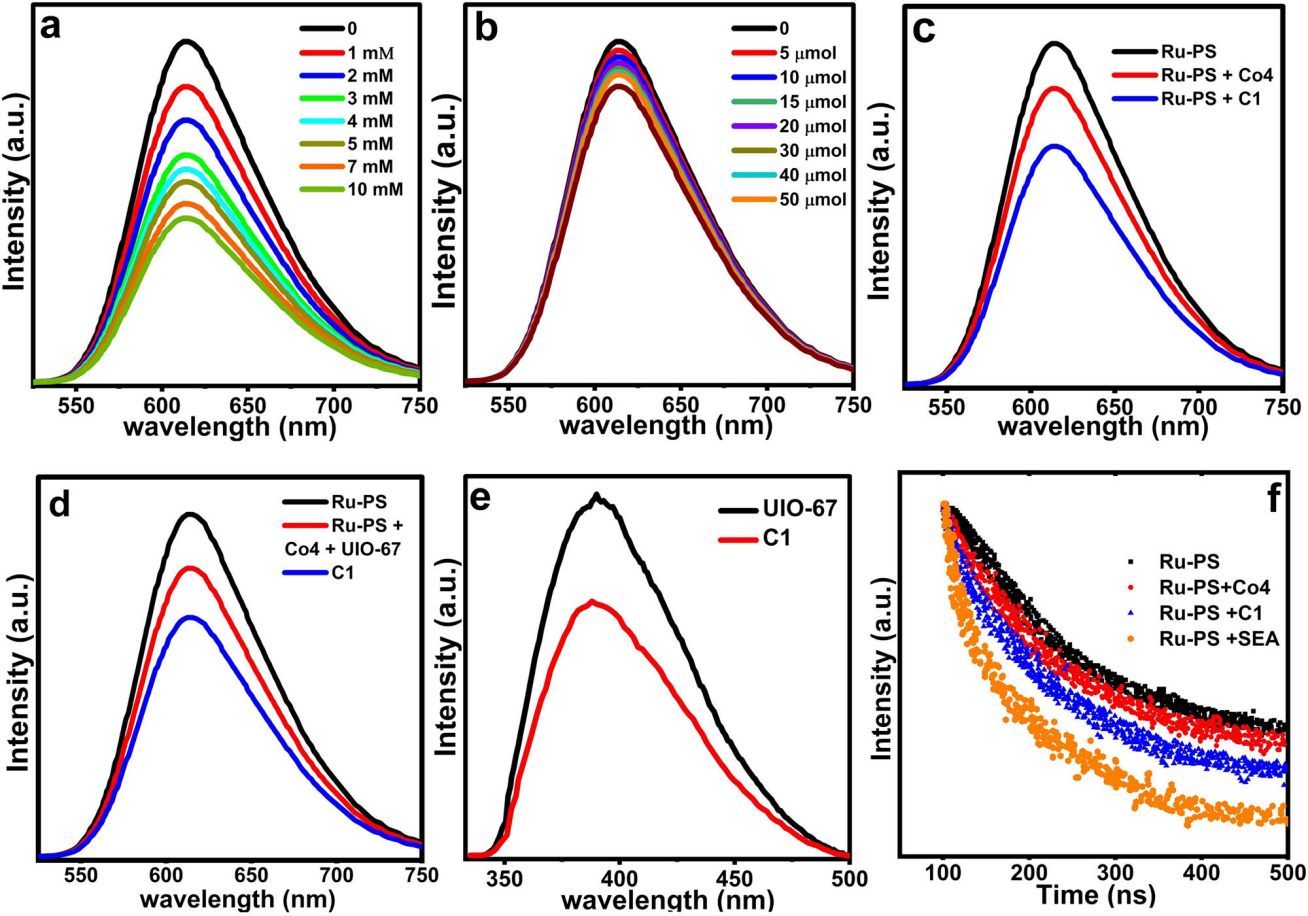
Photoluminescence studies of homogeneous and heterogeneous Co4. **a** PL of PS and various amounts of Co4. **b** PS and various amounts of SEA. **c** PS with Co4 and C1. **d** PS with C1, and a mixture of Co4 + UiO-67 mixture. **e** PL of UiO-67 and C1 (λex:300 nm). **f** Lifetime decay of Pure Ru-PS, and in the presence of Co4, C1, and SEA.

PL analysis of the PS exhibits a characteristic emission peak^46^. Upon introducing C1 or Na₂S₂O₈ (SEA), a compelling quenching behavior was observed. Since the excited state of PS can be quenched either reductively by C1 or oxidatively by SEA, identifying the dominant quenching pathway was essential^47^. To probe the initial electron transfer step, PL quenching experiments were performed by (i) varying the concentration of C1 in the presence of constant SEA, and (ii) varying SEA concentration with constant C1, while keeping all other conditions identical. As illustrated in Fig. 5a, b, PS luminescence was quenched significantly more by SEA than by C1. This strongly supports an oxidative quenching mechanism—wherein SEA accepts electrons from the excited PS—rather than a reductive pathway via C1. These observations are consistent with earlier studies on water oxidation systems^48,49^.

Previous investigations have established that sluggish electron transfer often limits catalytic performance, as it cannot effectively compete with rapid intrinsic excited-state relaxation. In homogeneous water splitting systems involving POMs, SEA/SED, and PS, ion-pair formation partially aids electron transfer, but its efficiency is restricted by component separation in solution. Encapsulation of POMs within MOFs offers a promising alternative, providing a confined microenvironment where Co4, PS, and SEA can co-exist in proximity. However, the cavity size plays a pivotal role—too small a cavity prevents co-localization of all components, while too large a cavity reduces contact and hampers interactions. Based on this rationale, it was hypothesized that an MOF like UiO-67, which offers sufficient space and interfacial contact area, could significantly enhance charge transfer efficiency and thereby catalytic activity^48^.

To validate this, PL quenching of PS was compared between homogeneous Co4 and encapsulated Co4 in UiO-67 (C1). As shown in Fig. 5c, C1 exhibited markedly stronger quenching, indicating more efficient electron transfer due to the spatial confinement and proximity of PS and Co4 within the MOF pores. This finding was further substantiated by comparing quenching efficiencies between C1 and a physical mixture of Co4 and UiO-67 (Fig. 5d). The physical mixture showed considerably weaker quenching, underscoring the benefit of true encapsulation. To evaluate any direct interaction between Co4 and UiO-67, PL spectra of bare UiO-67 and C1 were compared (Fig. 5e). The reduced emission in C1 suggests a strong interaction between the POM and the MOF framework.

### Stern–Volmer analysis

To further investigate the dominant quenching mechanism in our system, the luminescence of the excited Ru-PS was measured as a function of both Co4 and SEA concentrations separately (Fig. 5a). Linear fitting of the Stern–Volmer plot yields apparent rate constants (Kq) of 8.4 × 10^9^ M^−1^ s^−1^ for reductive quenching by Co4 and 5.3 × 10^8^ M^−1^ s^−1^ for SEA (Fig. 5b and Fig. S8). Although the rate constant for reductive quenching by Co4 is higher than that for SEA, the oxidative quenching process remains dominant due to the much higher concentration of SEA (10 mM) compared to Co4 (50 μM). The observed rate constants (K_obs_) for the reaction were calculated based on the concentration of the quencher (K_obs_ = Kq[Q]). We obtained values of 4.2 × 10^5^ and 3.7 × 10^6^ M^−^^1^ s^−1^ for Co4 and SEA, respectively, confirming that oxidative quenching is faster and dominates the overall process^50,51^.

### Time-resolved photoluminescence measurements

Time-resolved fluorescence spectroscopy was employed to monitor the luminescence decay of Ru-PS*(Fig. 5f). The excited-state lifetime of PS was recorded at 371 ns upon excitation at 450 nm. In the presence of SEA, Co4, and C1, the lifetime decreased to 230 ns, 337 ns, and 311 ns, respectively^47,48^. This confirms that oxidative quenching dominates over reductive quenching. Notably, the quenching of excited-state lifetimes, especially for C1, indicates more efficient charge separation and faster electron transfer dynamics compared to Co4. These findings further validate that the encapsulation of Co4 within UiO-67 enhances photocatalytic activity by improving electron mobility within the system.

### Proposed mechanism

**Scheme 1:**
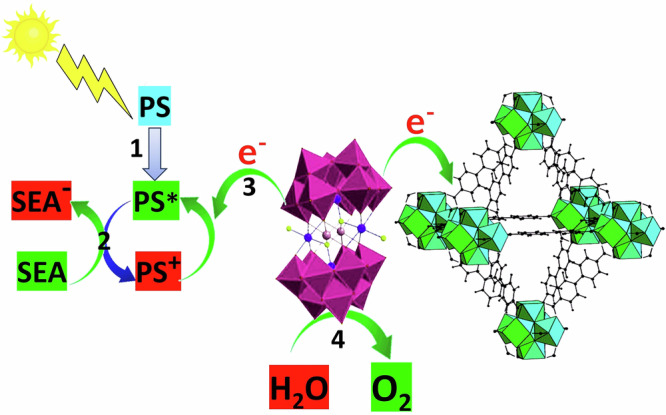
Mechanism of OER by C1.

The catalytic cycle begins with water molecules adsorbing onto the Co4 POM surface via hydrogen bonding, a well-established interaction for polyoxometalates as illustrated in Scheme 1^52–54^. This association facilitates efficient electron transfer from the water molecules to the POM, and such coordination at the active site is considered a critical first step in successful water oxidation catalysis^55^.

Drawing upon previous studies involving PMOF composites for water oxidation, along with our experimental observations of electron transfer behavior among POMs, MOFs, photosensitizers (PS), and SEA, we propose the following mechanistic steps^15,56–58^. (1) The photosensitizer absorbs visible light and transitions to its excited state. (2) The excited PS is oxidized by SEA, generating an oxidized PS species. (3) The oxidized PS then oxidizes Co4. (4) Finally, Co4 oxidizes water to release molecular O₂^15,16^.

Simultaneously, electron transfer from Co4 to the UiO-67 framework also occurs, as supported by our XPS, PL data, and corroborated by prior studies. The ability of MOFs like UiO-67 to accept electrons from POMs is well established and enhances the catalytic potential of Co₄ by promoting stronger water molecule adsorption and oxidation at its active site^44,59^. These enhancements in POM reactivity upon encapsulation in MOFs are widely documented in earlier literature^7,60–62^.

### Explanation for heightened activity

Previous studies have shown that encapsulating K₁₀[(PW₉O₃₄)₂Ni₄(H₂O)₂] (Ni₄P₂) in UiO-67 suppresses its hydrogen evolution reaction (HER) activity due to the framework’s ability to withdraw electronic density from Ni₄P₂, thereby diminishing its proton reduction capability. Here, however, this unique electronic interaction of UiO-67 is cleverly exploited: UiO-67 accepts electronic density from Co4, as clearly evidenced by XPS and photoluminescence (PL) results, enabling Co4 to more effectively drive water oxidation.

Compared to the naked POM, Co4 encapsulated within UiO-67 can efficiently dissipate electrons generated during water oxidation not only to the SEA but also to the MOF itself. XPS analysis reveals enhanced orbital overlap between Co4 and UiO-67, indicating superior electronic communication and more efficient electron transfer pathways within the composite.

Among various Co4@MOF composites, the exceptional “size-match” confinement of Co4 inside UiO-67’s pores creates an intimate fit that maximizes the contact area between the POM and the framework. This enhanced interface significantly accelerates electron transfer kinetics, leading to superior catalytic activity.

UiO-67’s cavities act as microreactors where all key components—POM, PS, and SEA—are confined in close proximity, unlike in homogeneous systems where these species are dispersed in solution. The dimensions of these cavities are critical: they must be sufficiently spacious to accommodate all reaction components and allow facile diffusion of reactants and products, while simultaneously providing extensive surface contact to promote electron transfer.

Immobilization of POMs within MOFs enhances their redox properties through strong electrostatic interactions, which translate into amplified catalytic performance^39^. This improved oxidizing power, coupled with increased electronegativity, strengthens hydrogen bonding between POMs and water molecules, enriching substrate concentration at the catalytic surface and facilitating the crucial initial adsorption step.

Water oxidation by Co4 begins with the attachment of water molecules to its active sites. The presence of UiO-67 intensifies this process because Co4 partially transfers electronic density to the MOF, making Co4 a superior adsorption site, thereby accelerating this rate-determining step^53,54^.

Solid-state UV-visible analysis further confirms that C1 exhibits enhanced light-harvesting capabilities compared to free Co4, boosting photocatalytic efficiency. Following photoexcitation of Co4 electrons from the valence band (VB) to the conduction band (CB), electron-hole recombination is effectively suppressed as the excited electrons are rapidly transferred to UiO-67^63^.

Previous research on water oxidation catalysts highlights that sluggish electron transfer rates often limit catalytic efficiency due to competition with excited-state relaxation. In contrast, C1 demonstrates significantly improved electron transfer kinetics—as supported by mechanistic studies—making it a key factor behind the remarkable enhancement in catalytic performance.

### Post-activity characterizations

To assess the stability of the composite during photocatalysis, a series of post-reaction characterizations was performed. PXRD patterns of the recovered C1 composite showed no discernible changes in the 2θ values compared to the fresh material, confirming that the UiO-67 framework remains structurally intact throughout the catalytic process (Fig. S9). Minor differences observed in the XRD patterns of the recycled catalyst are a common phenomenon in POM@MOF composites, as reported in previous studies, and are likely attributed to a slight decrease in long-range crystalline order, possibly due to partial amorphization^4^. Additionally, slight distortions in the MOF framework upon accommodating POMs have been reported, as the MOF adjusts to properly host the POMs^64–66^.

UiO-67 is known for its remarkable resilience, capable of withstanding aqueous environments ranging from strongly acidic to highly basic conditions^67^. This stability is primarily due to its composition: zirconium (IV) oxocluster nodes and linear dicarboxylate linkers. The high oxidation state of Zr(IV) contributes significantly to the exceptional stability of UiO-67, with Zr(IV) acting as a hard acid and carboxylate ligands as hard bases, forming strong coordination bonds. These Zr–O bonds give UiO-67 its outstanding chemical, thermal (up to 500 °C), and mechanical stability across a wide pH range. While some studies report the hydrolysis of UiO-67 under highly acidic (<2) or highly basic (>12) conditions, it remains stable under neutral or mildly acidic/basic environments. Since our experiments were conducted at pH 7–8, we can confidently rule out any risk of hydrolysis. Furthermore, studies show that the primary cause of structural degradation is mechanical stress during solvent removal, caused by capillary forces during water evaporation. This issue can be avoided by exchanging water with a less polar solvent (e.g., THF) before drying, ensuring no structural collapse^67^. In our case, the composite cavities, nearly filled with POMs that match the cavity size, eliminate any concerns of structural collapse. Moreover, it has been well established that POMs within MOFs act as pillars, enhancing the framework’s strength and stability. Numerous studies provide substantial evidence that the incorporation of POMs into MOFs significantly improves their stability and structural integrity^64,68,69^.

To further verify the catalyst’s stability, we measured the BET surface area of the recycled catalyst. The surface area remained almost identical to that of the pristine material, providing clear evidence that no structural collapse occurred (Fig. S10). This stability is consistent with other studies on POM@UiO-67 composites, where BET analysis shows little to no change after catalytic reactions^45^.

To confirm the persistent composition and oxidation states of the elements, XPS analysis was performed on C1 after photocatalytic activity (Fig. S11). The results showed no significant shifts in the chemical states or elemental composition, reinforcing that Co4 is robustly retained within the UiO-67 matrix, maintaining its chemical integrity under reaction conditions. Finally, the intact morphology of C1 was further confirmed through TEM analysis, which showed that the size and shape of the particles remained unchanged before and after catalysis (Fig. S12).

Altogether, these results provide compelling evidence of the catalyst’s exceptional stability under catalytic conditions, ruling out any possibility of structural collapse or decomposition during recyclization.

## Conclusion

We successfully synthesized a novel composite, Co4@UiO-67 (C1), by encapsulating the noble-metal-free polyoxometalate Co4 within the stable Zr(IV)-based MOF UiO-67. Extensive characterization confirmed stable incorporation of Co4 inside the MOF cavities, excluding surface adsorption, ensuring a true heterogeneous structure. C1 exhibits significantly enhanced photocatalytic OER activity over a wider pH range than homogeneous Co4 and outperforms previous Co4@MOF composites. This improvement arises from strong electrostatic interactions, increased hydrolytic and thermal stability, better light harvesting, enhanced electron transfer kinetics via a size-matched, leaching-resistant assembly, and higher electrophilicity of Co4 promoting water adsorption. The catalyst shows excellent recyclability, maintaining activity over multiple cycles without detectable leaching, as confirmed by leaching tests, PXRD, and XPS. The confinement within UiO-67 creates a microreactor environment, ensuring proximity and efficient interaction among Co4, photosensitizer, and sacrificial electron acceptor, optimizing electron transfer and catalytic efficiency.

This work advances understanding of electron transfer in PMOF composites and presents a versatile, leaching-resistant platform for noble-metal-free water oxidation. The Co4@UiO-67 composite demonstrates how precise control of pore size, framework stability, and electronic interactions can design efficient, durable photocatalysts for sustainable energy applications.

## Methods

### Materials and reagents

Details of materials and reagents, and synthesis of UiO-67 and Co4 are given in the Supplementary Information (SI).

### Synthesis of Co4@UiO-67 (C1)

The composite C1 was synthesized via a solvothermal method following the same procedure as for pristine UiO-67, with the only modification being the addition of 0.5 g of Co4 to the reaction mixture. After completion of the reaction, the resulting light purple solid was isolated by centrifugation and sequentially washed with water, acetone, and diethyl ether to remove unreacted precursors and surface-adhered species. The purified product was then dried in an oven at 80 °C overnight^15^. Yield: 61.5%.

### Leaching test

To check the stability of composite C1 and the retention of POMs inside the UiO-67 framework, leaching tests were performed. Typically, 5 mg of the composite was immersed in 25 mL of distilled water, and samples were taken from the supernatant after 1 h, 3 h, 5 h, 24 h, and 72 h. The UV–Vis spectra of these samples were recorded in the range of 380–650 nm. To detect any possible leaching of Co²⁺ ions from Co₄ or Zr⁴⁺ ions from UiO-67, the supernatants were also analyzed by AAS spectroscopy, both before and after the catalytic experiments. The UV–Vis spectra showed no characteristic absorption peaks, instead displaying a flat baseline throughout the measurement range. Similarly, AAS analysis revealed no detectable presence of Co²⁺ or Zr⁴⁺ in any of the filtrates. These results clearly confirmed that neither POMs nor metal ions leached from the composite, supporting the anticipated stability based on the narrow pore windows of UiO-67 and its excellent chemical robustness^19^.

### Photochemical experiments

All photocatalytic experiments were carried out at room temperature in a 25 mL reaction vessel sealed with a rubber septum, using a 5 µM concentration of the catalyst. Reaction conditions varied depending on the catalyst used. For oxygen evolution reaction (OER) with either Co4 or C1, a 10 mL sodium borate buffer (30 mM, pH 7.5–8.0) was employed, containing 5 mM Na₂S₂O₈ as a sacrificial electron acceptor. A 1 mM aqueous solution of [Ru(bpy)₃]Cl₂·6H₂O was used as the photosensitizer. The reaction mixtures were continuously stirred and irradiated using LED light (λ = 460 nm, 300 W). Evolved oxygen gas was monitored and quantified using a Shimadzu GC-2014 gas chromatograph equipped with a thermal conductivity detector (TCD). O₂ TONs were calculated using the following equations.

$${TON}=\frac{n[O2]}{{{\rm{n}}}[{{\rm{catalyst}}}]}$$

### Recovery of the catalyst

After irradiation during the photocatalytic reactions, the catalyst was recovered from the solution by centrifugation. The recovered solid was immersed in water and sonicated for 20 min to remove any adsorbed materials from the reaction mixture. After washing with water several times, the catalyst was dried under vacuum at room temperature prior to recycling experiments and post-catalytic characterizations.

### Determination of acidic proton

An established acid–base titration method was employed to determine the number of acidic protons in all composites^33,39^. Briefly, 0.1 g of the catalyst C1 was added to 25 mL of 0.0085 M NaOH solution, previously standardized using 0.0098 M potassium hydrogen phthalate. The mixture was sealed and stirred at room temperature for 12 h. After stirring, the suspension was filtered, and the filtrate was back-titrated with 0.0136 M HCl, which had been standardized with NaOH. The number of acidic protons was calculated based on the amount of NaOH consumed during the titration.

## Supplementary information

Supplementary Information

## Data Availability

All principal data with detailed experimental procedure and characterization of this work are included in this article and its Supplementary Information.
