# Supplementary material for: Precise size-matching between guest polyoxometalates and host metal-organic frameworks enables enhanced photocatalytic water oxidation
Source: Commun Chem. 2025 Dec 10;9:29. doi: 10.1038/s42004-025-01838-y (PMC12820358; doi:10.1038/s42004-025-01838-y)
Supplement: Supplementary file 1 — Supplementary Information [file 42004_2025_1838_MOESM1_ESM.pdf]

Supporting Information

Precise size-matching between guest polyoxometalates and host metal-organic frameworks enables enhanced photocatalytic water oxidation

Waqas Ali Shah[1], Xusheng Dai[1], Xiaowei Zhai[1], Yuanyuan Zhao[1], Yalei Zhang[1], Shujun Li[1]*

[1]School of Chemistry and Chemical Engineering, Henan Key Laboratory of Boron Chemistry and Advanced Materials, Key Laboratory of Green Chemical Media and Reactions, Ministry of Education, Collaborative Innovation Centre of Henan Province for Green Manufacturing of Fine Chemicals, Henan Normal University, Xinxiang, 453007, China. E-mail: lisj@htu.edu.cn

Materials and Reagents

Zirconium tetrachloride, Sodium tungstate dihydrate, Cobalt nitrate hexahydrate, nickel nitrate hexahydrate, disodium biphosphate heptahydrate, germanium oxide, benzoic acid, biphenyl-dicarboxylic acid, ($[Ru(bpy)_3]Cl_2·6H_2O$, 99.95%), sodium chloride, sodium hydroxide, sodium acetate, acetic acid, diethyl ether, Hydrochloric acid, $Na_2S_2O_8$, KNO_3, dimethyl formamide and acetone were purchased from Sigma Aldrich. All chemicals were used without further purification.

Supplementary methods

Synthesis of UIO-67

UiO-67 was synthesized according to the method given in the literature [1]. In 40 mL of DMF, $ZrCl_4$ (245 mg, 1.05 mmol), glacial acetic acid (2 mL) and biphenyl-dicarboxylic acid (260 mg, 1 mmol) were added and dissolved. 83 μL of 37 % HCl was added and the mixture was then stirred and mixed under ultrasonic for 30 min. at 120 °C for an hour. The resulting mixture was heated at 120 °C for a period of 24 h and then cooled to room temperature. The solid mixture was washed with DMF three times soaked in methanol for 48 hours. Finally, white solid of UIO-67 was obtained which was dried under vacuum. Yield: 57% based on zirconium.

Synthesis of Co4

Co4 was synthesized following a reported method [2]. Briefly, $Na_2HPO_4·7H_2O$ (3.22 g, 12 mmol), $Na_2WO_4·2H_2O$ (35.62 g, 108 mmol), and $Co(NO_3)_2·6H_2O$ (6.98 g, 24 mmol) were mixed in 25 mL of deionized water producing a solution. The pH of the suspension was adjusted to 7 by adding an HCl solution to it followed by refluxing it at 100 ºC for 2 hrs. The solution was then saturated by adding NaCl and allowed to cool to room temperature. The resulting violet crystals of the product were separated and quickly washed several times with deionized water and finally recrystallized using hot water. Yield: 63% based on cobalt.

Characterizations

Powder X-ray diffraction (XRD) diffractograms were recorded at room temperature on a diffractometer (Bruker AXS D8, 20 mA and Cu Kα, = 1.5406 Å, 40 kV) in the range of 2θ = 5 to 40°. While, the simulated patterns of XRD were calculated using single-crystal X-ray diffraction data form Cambridge Crystallographic Data Center (CCDC). FT-IR spectra of KBr pellets were obtained with a AUTOLAB in the range of 1800–400 cm^{-1}. The nitrogen adsorption studies were carried out on micromeritics ASAP 2020. Before adsorption and desorption studies, all samples were degassed in vacuum for 12 h at 150 °C. Thermogravimetric analysis (TGA) was conducted in air atmosphere (60 mL/min) using a Perkin Elmer electrobalance TGA-7 at the heating rate of 10 °C per min. Elemental Analysis was performed on ICP-MS: Agilent 7800 analyzer. UV-vis spectra were collected with an Agilent 8453 UV-vis diode array spectrophotometer and Agilent ChemStation software using a spectral bandwidth of 1.0 nm. Samples were measured in a quartz cuvette with a 1 cm path length were used for the measurements of samples. The composition analysis was conducted with XPS measurements under ultrahigh vacuum (UHV) with a Kato, ESCALLAB 250Xi monochromatized Al Kα cathode source, using a low energy electron gun for charge neutralization. The morphological and structural examination and elemental mapping were accompanied with transmission electron microscopy (TEM, JEOL, JEM-2100F) and energy-dispersive X-ray (EDX) spectroscopy.

Table S1. Photocatalytic activity of various compositions of C1 with various loading amounts of Co4 in UiO-67

**Name**	**Formula**	**POMs (%)**	**O_2 TONs**
**1**	$[H_{10}(PW_9O_{34})_2Co_4(H_2O)_2]_{0.1}$@ UIO-67	16.8	91
**2**	$[H_{10}(PW_9O_{34})_2Co_4(H_2O)_2]_{0.15}$@ UIO-67	23.23	167
**3**	$[H_{10}(PW_9O_{34})_2Co_4(H_2O)_2]_{0.19}$@ UIO-67	27.71	236
**4** (C1)	$[H_{10}(PW_9O_{34})_2Co_4(H_2O)_2]_{0.21}$@ UIO-67	29.7	253
**5**	$[H_{10}(PW_9O_{34})_2Co_4(H_2O)_2]_{0.23}$@ UIO-67	31.8	214

5 µM catalyst's concentration, with 5 mM SEA, and 1 mM PS in 30 mM borate buffer at various pH

Table S2. Elemental analysis of UIO-67, Co4 and the composite C1

Elements	UIO-67	Co4	C1
**Zr**	24.9	--	15.7
**C**	44.3	--	30
**Co**	--	4.46	1.42
**P**	--	1.17	0.37
**W**	--	62.50	20
**Na**	--	4.34	--

Table S3. Formulae and other compositional parameters of the composites calculated from of ICP and TGA .

Name	Formula	MW (g)	MOF (g/%)	POMs (g/%)	Organic (g/%)	H_2O (g/%)	Total weight loss (%)
**UIO-67**	$[Zr_6O_4(OH)_{5.4}][C_{14}H_8O_4]_{5.3}$	2191.32	2191/ 100	0/0	75	0	65.2
**Co4**	$Na_{10}[(PW_9O_{34})_2Co_4(H_2O)_2]$. $20H_2O$	5290.34	0/0	5290.34 / 100	0/0	6.8	--
**C1**	$[H_{10}(PW_9O_{34})_2Co_4(H_2O)_2]_{0.21}$ @ UIO-67. $14H_2O$	3478.69	2191.32 /63	986.65/ 29.7	1644/ 47.2	7.2	41.5

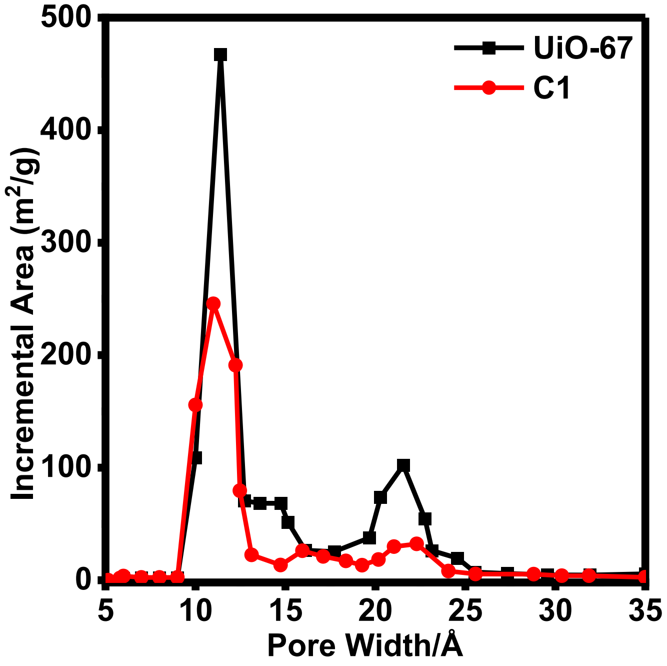

Figure S1. Pore size distribution (PSD) profiles of UiO-67 and C1

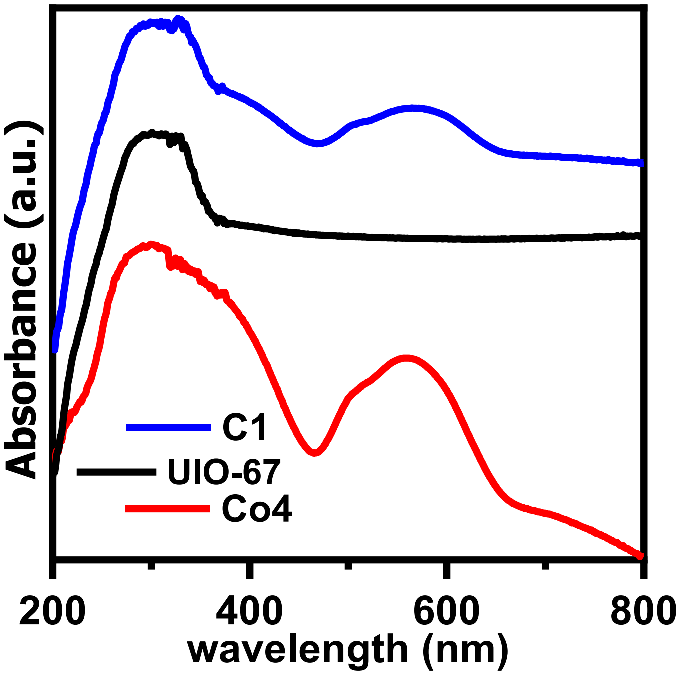

Figure S2. Solid-state UV-Visible spectra of **Co4**, UIO-67 and **C1**

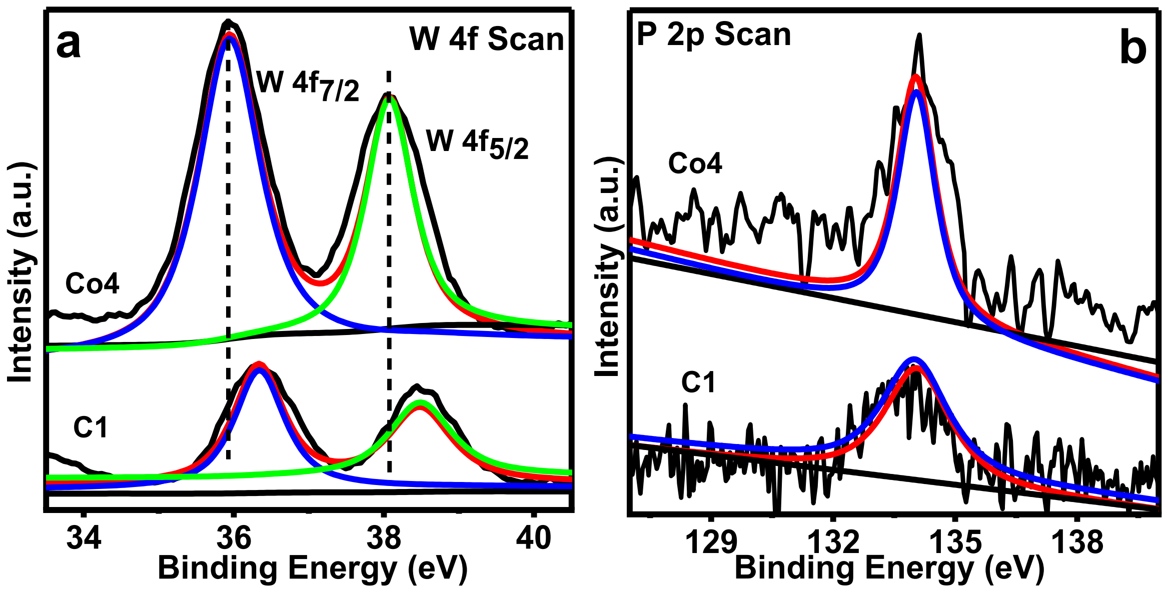

Figure S3. XPS of (a) W in Co4 and C1 (b) P in Co4 and C1

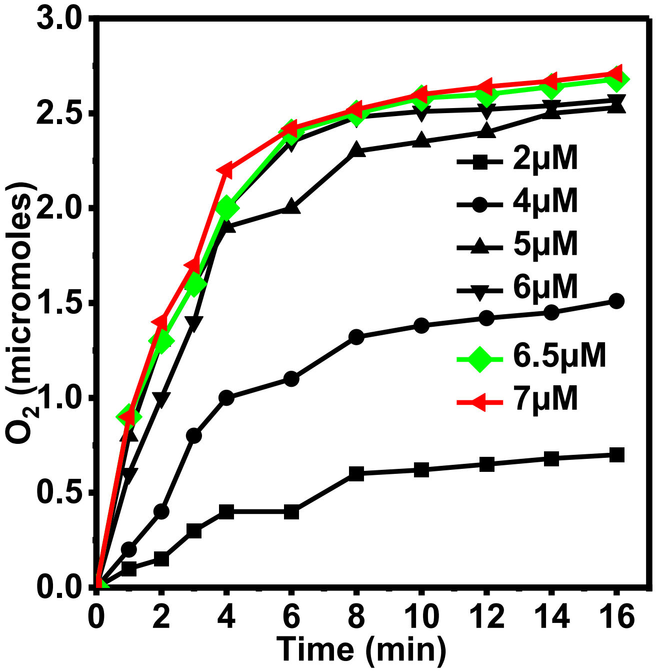

Figure S4. OER by various concentration of C1 with 5 mM SEA, 1mM PS, 30 mM borate buffer at pH 8.

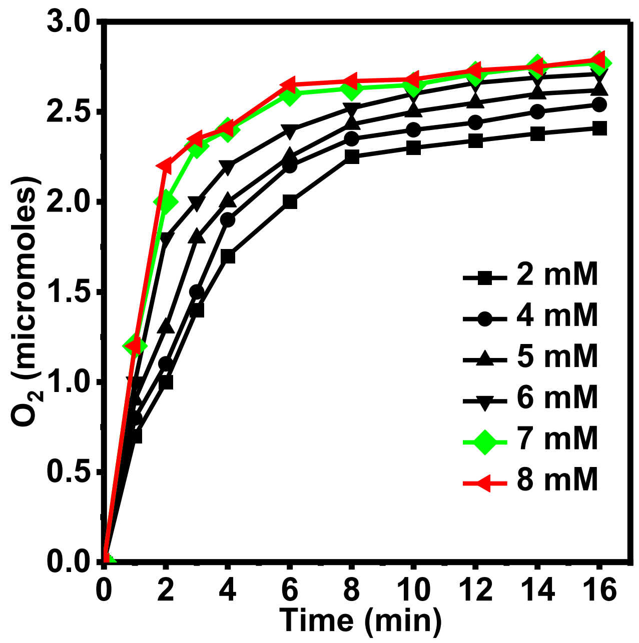

Figure S5. OER by 6.5 µM C1 with 1mM PS, 30 mM borate buffer at pH 8 with various concentration of SEA

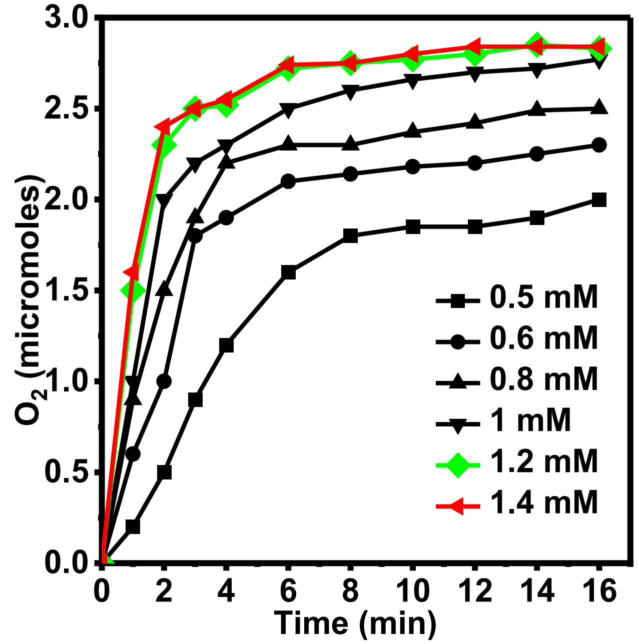

Figure S6. OER by 6.5 µM C1, 7 mM SEA, 30 mM borate buffer at pH 8 with various concentration of PS

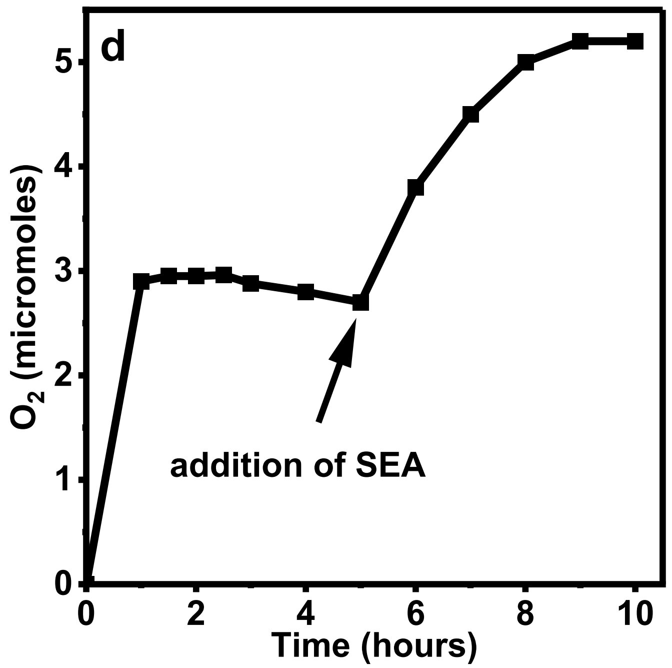

Figure S7. Long-term activity of C1 using 6.5 μM catalyst's concentration, with 7 mM SEA, and 1.2 mM PS in 30 mM borate buffer at pH 8

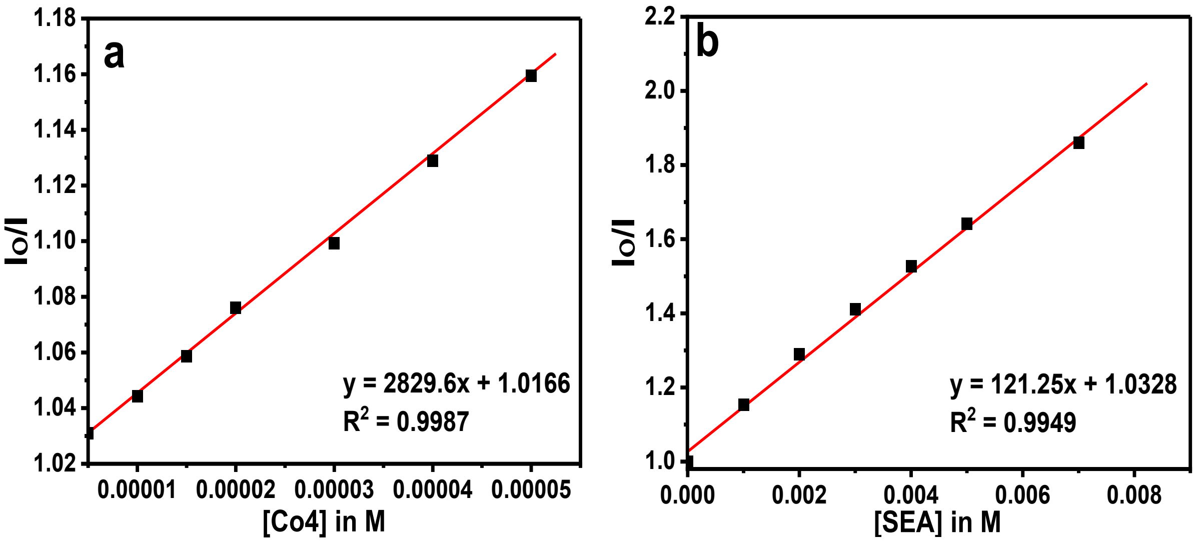

Figure S8. Stern-Volmer plots for quenching of Ru-PS by Co4 and SEA

Post-activity characterizations

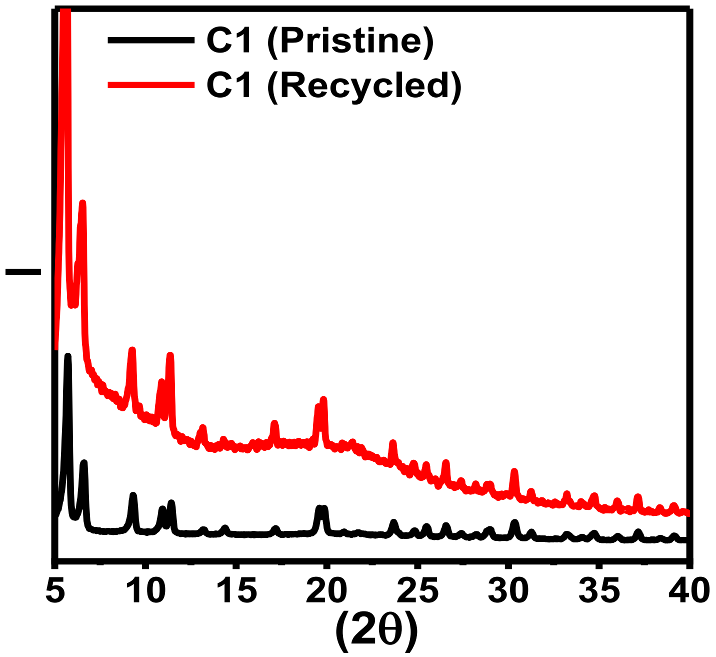

Figure S9. PXRD of pristine and recycled C1

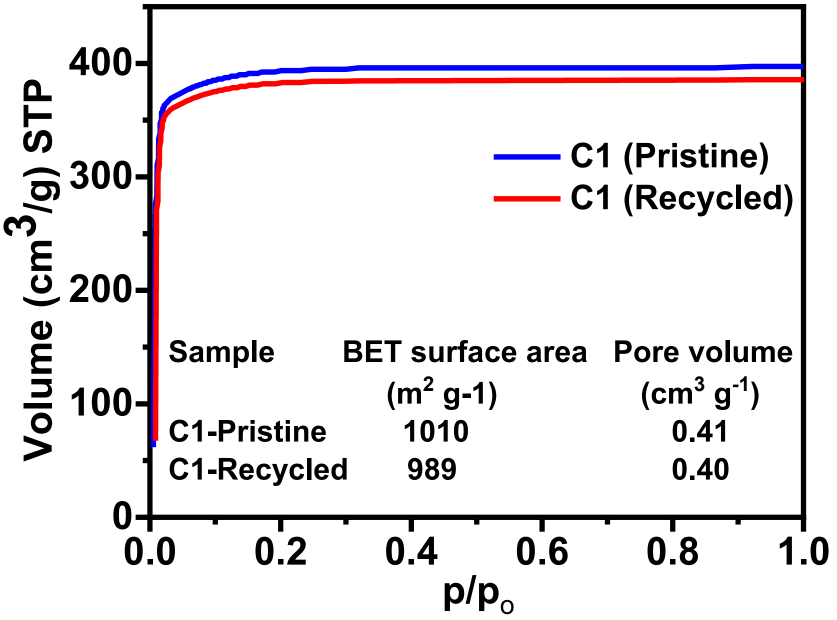

Figure S10. BET analysis of pristine and recycled C1

Table S4. Surface area and porosity ratio measurements for UiO-67, and C1 (before and after catalysis)

**Sample**	**BET surface area (m^2 g^{-1})**	**Pore volume (cm^3 g^{-1})**
UiO-67	2110	0.89
**C1**	1010	0.41
**C1**-Recycled	989	0.40

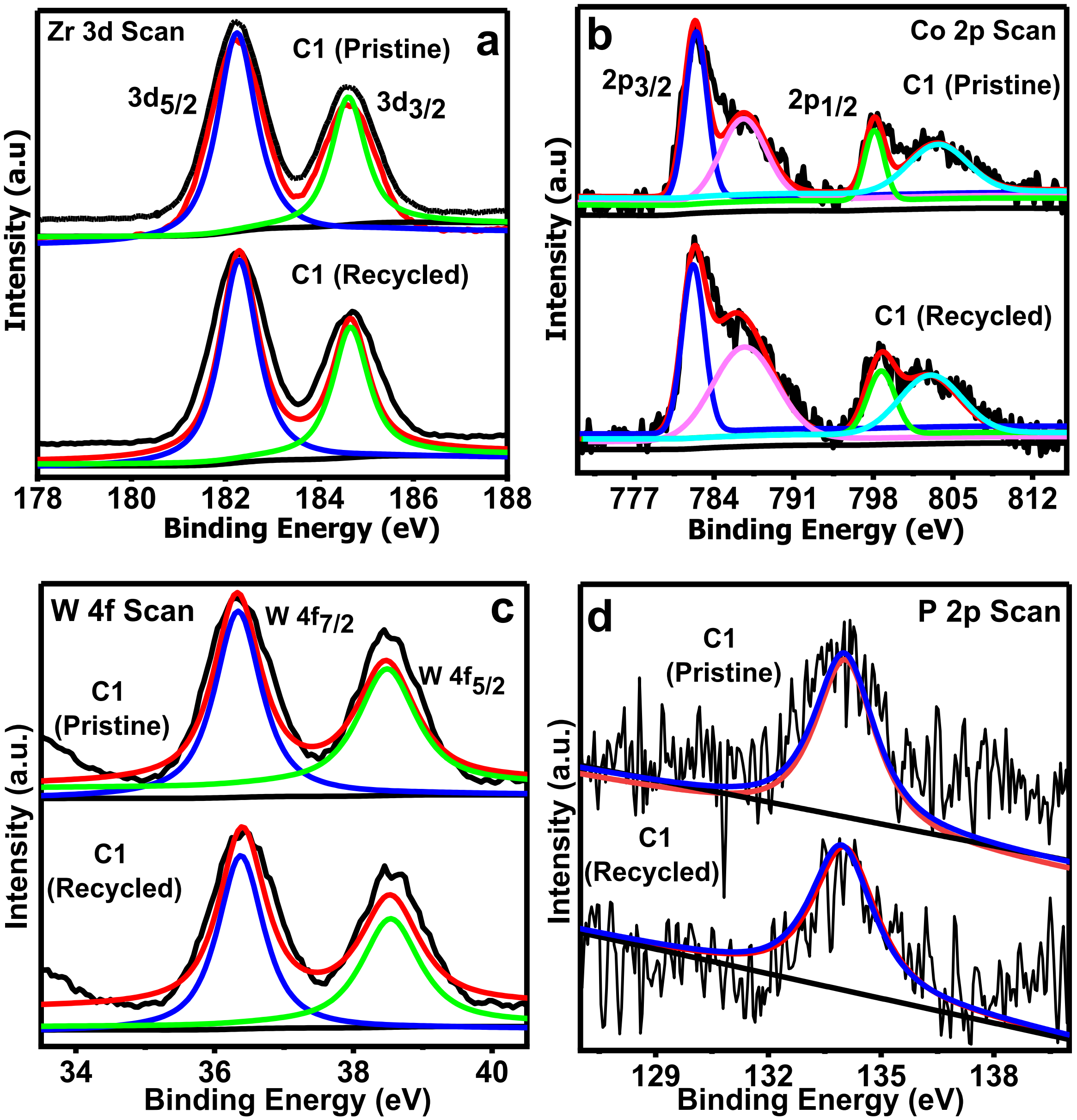

Figure S11. XPS of pristine and recycled C1 (a) Zr (b) Co (c) W (d) P

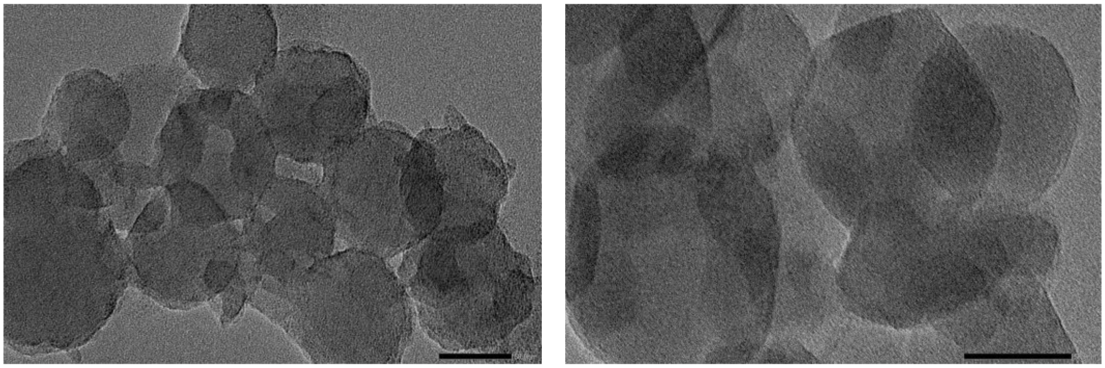

Figure S12. TEM of recycled **C1** at 100 nm (left) and 50nm (right)

Supplementary References

1. Cavka, J.H., S. Jakobsen, U. Olsbye, N. Guillou, C. Lamberti, S. Bordiga and K.P.J.J.o.t.A.C.S. Lillerud, A new zirconium inorganic building brick forming metal organic frameworks with exceptional stability. *Journal of the American Chemical Society*, 2008. **130**(42): p. 13850-13861.
2. Yin, Q., J.M. Tan, C. Besson, Y.V. Geletii, D.G. Musaev, A.E. Kuznetsov, Z. Luo, K.I. Hardcastle and C.L. Hill, A fast soluble carbon-free molecular water oxidation catalyst based on abundant metals. *Science*, 2010. **328**(5976): 342-353.
